# Voltage Amplifier Based on Organic Electrochemical Transistor

**DOI:** 10.1002/advs.201600247

**Published:** 2016-09-17

**Authors:** Marcel Braendlein, Thomas Lonjaret, Pierre Leleux, Jean‐Michel Badier, George G. Malliaras

**Affiliations:** ^1^ Department of BioelectronicsEcole Nationale Supérieure des MinesCMP‐EMSE, MOCGardanne13541France; ^2^ MicroVitae TechnologiesHôtel TechnologiqueMeyreuil13590France; ^3^ Institut de Neurosciences des SystèmesAix‐Marseille Université, INS/Inserm13005MarseilleFrance

**Keywords:** device physics, electrocardiography, organic electronics, poly(3,4‐ethylenedioxythiophene):poly(styrene sulfonate), voltage amplifier circuit

## Abstract

Organic electrochemical transistors (OECTs) are receiving a great deal of attention as amplifying transducers for electrophysiology. A key limitation of this type of transistors, however, lies in the fact that their output is a current, while most electrophysiology equipment requires a voltage input. A simple circuit is built and modeled that uses a drain resistor to produce a voltage output. It is shown that operating the OECT in the saturation regime provides increased sensitivity while maintaining a linear signal transduction. It is demonstrated that this circuit provides high quality recordings of the human heart using readily available electrophysiology equipment, paving the way for the use of OECTs in the clinic.

## 1 Introduction

Historically, electrophysiological activity has been recorded with amplifiers using cutaneous or implanted electrodes to pick up the biological signal and dates back to the work of Einthoven in 1901.^1^ The displacement of ionic charge inside biological tissues during heart or neuronal activity builds up a voltage which can be read with low impedance electrodes.^2^ However, on the way to the amplifier, this biological information is disturbed by ambient noise such as the characteristic AC signal from the building's power supply.^3^ This can become particularly challenging in environments such as an operating theater in a hospital where strong lights, space limitations, and sophisticated medical tools provide high electrical noise. This is compounded by the fact that the amplitude of electrophysiological signals is typically very low. One way to bypass such problems is to use active transducing amplifiers that are in direct contact with the biological medium. This provides a first stage amplification at the recording site, making the signal less sensitive to further noise pickup.

A promising candidate for such a transducing amplifier is the electrolyte gated organic electrochemical transistor (OECT), a three terminal device with key properties such as low operation voltage, high transconductance, and biocompatibility.^4^, ^5^, ^6^, ^7^ The use of the conducting polymer poly(3,4‐ethylenedioxythiophene) doped with poly(styrene sulfonate) (PEDOT:PSS) as active material leads to an improved bioelectronic interface and recent studies have attributed this fact to the mixed ionic and electronic conduction where ions are able to penetrate into the bulk of the material.^8^, ^9^, ^10^ Easy fabrication with low temperature processing techniques, such as photolithography, screen printing, or inkjet printing, provide a future perspective for large scale manufacturing of flexible, disposable, and reasonably priced sensor devices on a broad range of substrates.^11^, ^12^, ^13^, ^14^, ^15^ As such, the OECT has proven worthy in various biosensing scenarios, e.g., *in vitro* detection of ions, specific metabolites and nucleotides, real‐time monitoring of barrier tissue integrity, and *in vivo* recordings of electrophysiological activity.^16^, ^17^, ^18^, ^19^, ^20^

The OECT in its regular configuration, i.e., a constant source–drain bias and a gate that is connected to the source and immersed in an electrolyte covering the active material, provides a drain current *I*_D_ that is modulated by the gate voltage *V*_GS_. Cations injected in the active material upon applying a positive gate voltage compensate sulfonate anions on the PSS chain, thus reducing the number of holes in the film by electrochemically dedoping the conducting polymer.^21^ Campana et al. demonstrated a direct cutaneous implementation of an OECT on a resorbable bioscaffold for transient applications in electrocardiographic (ECG) recordings.^22^ This way, the amplifying transducer is brought directly to the site of interest, reducing the power‐line interference and allowing for an increased signal‐to‐noise ratio comparable to regular electrode measurements. In order to maintain high quality recordings for long‐term measurements, as needed for example in sports applications or telemedicine situations, hydrogels or ionic liquids gels are used to establish a better contact of electrophysiology devices with the skin.^23^, ^24^, ^25^, ^26^ This reduces motion artifacts and maintains or lowers the impedance.^27^

To make the OECT compatible with existing voltage recording equipment for electrophysiological measurements, we need to convert the current output into a voltage signal. In this article, we focus on the integration of the OECT into a simple voltage amplifier circuit to provide a voltage‐to‐voltage transduction, as first introduced by Rivnay et al.^28^ A detailed investigation of this system allows us to optimize the amplification parameters of the device. We find an increased performance when biasing the OECT in the saturation regime with a linear amplification of up to 30 V/V. To validate our system, we demonstrate recordings of electrocardiographic signals with a regular medical recording system. This work leads to a better understanding of OECT‐based circuits and paves the way for their use as active organic electrodes with traditional electrophysiology instrumentation.

## 2 Results and Discussion

**Figure 1:**
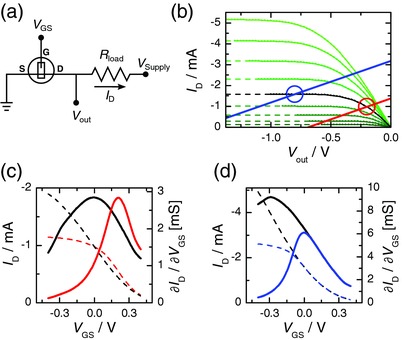
a) Schematic circuit layout of the voltage amplifier system with embedded OECT. b) Load line diagram of an OECT with aspect ratio of *W*/*L* = 2 and thickness of *d* = 70 nm using a drain load resistor of *R*_load_ = 500 Ω for saturation regime (blue) and linear regime (red). A 100 mm solution of sodium chloride is used as electrolyte. The gate voltage is varied from *V*_GS_ = +0.4 V (light green) to −0.4 V (dark green) in steps of ∆*V*_GS_ = 0.1 V. The black line indicates the output at *V*_GS_ = 0 V and the open circles denote the corresponding operation point. The dashed lines are a guide to the eye and extend the saturation regime to the inaccessible data points assuming that the drain currents fully saturates. c–d) Transfer curve (dashed line) and transconductance (solid line) of the unloaded (black) versus the loaded OECT (colored) for linear regime c) and saturation regime d). The values of the drain current are extracted graphically via the intersection of the load line with the output curve at each value of *V*_GS_.

To convert the current provided by the OECT into a readable voltage signal *V*_out_ we use a load resistor *R*_load_ on the drain side of the OECT (see **Figure** **1**a). This acts as a voltage divider for the supply voltage *V*_supply_ and lets the drain voltage *V*_out_ of the OECT float, i.e., there is no fixed source–drain bias anymore. Instead, the bias depends on the drain current

(1) $$V_{\mathrm{out}}=V_{\mathrm{supply}}-R_{\mathrm{load}}\cdot I_{D}\left(V_{\mathrm{GS}},V_{\mathrm{out}}\right)$$

which itself depends on both the gate voltage as well as the source–drain bias. Therefore, a modulation on the gate can be related to a change in the output voltage and a voltage‐to‐voltage transduction is achieved. For each gate voltage there is one specific output voltage and this so called operation point (OP) depends on the drain load as well as the supply voltage. By solving Equation (1) for *I*_D_ and plotting this so called load line on top of the IV curve one obtains a load line diagram and can immediately extract the operation point at the intersection of the two curves (see Figure 1b). Since the effective source–drain bias of the device varies linearly with the drain current, the resulting transconductance *g*_m_ = ∂*I*_D_/∂*V*_GS_ is also altered. We distinguish between the linear and the saturation regime of the OECT, the chosen operation points at *V*_GS_ = 0 V are OP_lin_ = −0.2 V and OP_sat_ = −0.8 V respectively (see Figure 1c,d). It can be seen that in the linear regime, the peak transconductance compared to a device without drain load is shifted toward a higher positive gate voltage and consequently the transconductance at *V*_GS_ = 0 V drops by more than 50%. On the other hand, in the saturation regime the transconductance is identical to an unloaded device for positive gate voltages and only deviates for negative gate voltages. This is due to the fact that at negative gate voltages, the drain current increases and in consequence the source–drain bias decreases until the OECT operates back in the linear regime. Considering the amplitude of regular electrophysiological signals (in the order of μV to mV), we are mainly interested in small changes of the gate voltage around *V*_GS_ = 0 V. Hence, operating the device in the saturation regime is favorable as it provides good transduction.

In analogy to the findings of Chou et al.,^29^ we can derive a relationship between the transconductance of the loaded and the unloaded device, using the differential form of the drain current

(2) $$\begin{array}{l} dI_{D}={\frac{\partial I_{D}}{\partial V_{\mathrm{GS}}}|}_{V_{\mathrm{out}}}\cdot dV_{\mathrm{GS}}\;+\;{\frac{\partial I_{D}}{\partial V_{\mathrm{out}}}|}_{V_{\mathrm{GS}}}\cdot dV_{\mathrm{out}}\\ =g_{m}^{i}\cdot dV_{\mathrm{GS}}+g_{d}^{i}\cdot dV_{\mathrm{out}} \end{array}$$

where *g* ^i^_m_ and *g* ^i^_d_ are the intrinsic transconduction and drain conductance respectively (i.e., with no drain load resistor). Using Equation (1) it follows that

(3) $$d\;I_{D}=-\frac{1}{R_{\mathrm{load}}}\cdot dV_{\mathrm{out}}$$

and Equation (2) can be solved for d*V*_out_/d*V*_GS_, i.e., the voltage gain of the system

(4) $$\mathrm{Gain}=\left|\frac{dV_{\mathrm{out}}}{dV_{\mathrm{GS}}}\right|\;=\left|\frac{g_{m}^{i}}{1/R_{\mathrm{load}}+g_{d}^{i}}\right|\;\;$$

**Figure 2:**
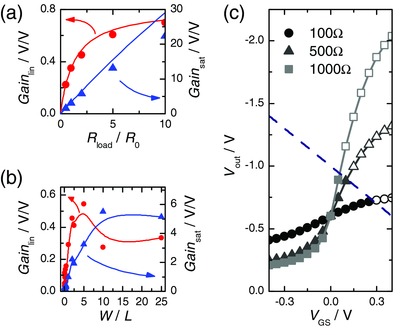
a) Voltage gain ∆*V*_out_/∆*V*_GS_ for different drain load resistors for an input signal of *∆V*_GS_ = 1 mV in both the linear regime (red, OP_lin_ = −0.2 V) and the saturation regime (blue, OP_sat_ = −0.8 V). The channel resistance is *R*_load_ = 213 Ω and *R*_0_ = 513 Ω for the linear regime and saturation regime, respectively. The solid line shows an analytical model according to Equation (4). The same device is used as in Figure 1. b) Voltage gain ∆*V*_out_/∆*V*_GS_ for different aspect ratios for a drain load resistor of *R*_load_ = 500 Ω in the linear regime (red) and the saturation regime (blue). The points are calculated according to Equation (4) using experimentally derived IV curves for each geometry. The thickness of the devices is *d* = 70 nm. The solid lines are a guide to the eye. c) Output voltage versus gate voltage for different drain load resistors for the same device as in Figure 1. The operation point at *V*_GS_ = 0 V is set to −0.6 V. The values are extracted graphically via the intersection of the load line with the output curve at each value of *V*_GS_. Open symbols denote data outside the experimental limitation of |*V*_GS_ – *V*_out_| < 1 V, indicated by the dashed blue line, that allows for stable operation of the device.

From this relation, it becomes clear that in the saturation regime, where the drain current is independent of the source–drain bias (i.e., *g* ^i^_d_ = 0), the voltage gain is directly proportional to the transconductance times the drain load resistor, whereas in the linear regime the voltage gain saturates for higher drain load as 1/*R*_load_ goes to zero (see **Figure** **2**a). We compare this model to experimental data that have been retrieved by generating a sinusoidal signal with an amplitude ∆*V*_GS_ = 1 mV and a frequency *f* = 2 Hz at the gate and recording the output voltage over a few cycles. We again distinguish between the linear and the saturation regime, meaning that we adjust *V*_out_ to obtain the given operation points (−0.2 V and −0.8 V) at *V*_GS_ = 0 V for each value of the drain load resistor. The model shows remarkable agreement with the experimental data. Only at high drain load the voltage gain is overestimated in the saturation regime. This can be explained with the fact that at such a high voltage gain (> 20 V/V), a small inevitable drift in the drain current that derives for example from evaporation of the electrolyte also gets amplified by the same amount and consequently the operation point at zero gate bias is shifted toward higher source–drain bias.

Using Equation (4), we can also check the effect of device geometry for a given operation point and drain load resistor by using the intrinsic transconductance and drain conductance extracted from an IV curve of the plain OECT (see Figure 2b). It can be seen that again an overall higher gain can be achieved in the saturation regime. In the linear regime, the maximum gain can be obtained for aspect ratios of about *W*/*L* = 5 and in the saturation regime it seems to be at around *W*/*L* = 15. Rivnay et al. showed that the transconductance at *V*_GS_ = 0 V increases for increasing aspect ratio until a certain point (for a channel thickness of *d* = 140 nm this is up to *W*/*L* ≈ 10, for *d* = 25 nm it is *W*/*L* > 10).^28^ This explains the findings in Figure 2b for the saturation regime as in this case the gain is directly proportional to the transconductance and the drain conductance can be neglected. In the linear regime, where the drain conductance is linear in *W*/*L*, the peak gain is shifted to lower aspect ratios as the drain conductance dominates in the denominator.

We emphasize the fact that this analytical derivation does not rely on any model for the OECT as it needs only the electrical characteristics (i.e., transconductance and drain conductance) of the plain transistor without any drain load. It is possible to arrive at an analytical expression and obtain similar findings using the Bernards model of the OECT.^30^ However, the agreement with the experimental data is not as good (see the Supporting Information) which can be mainly attributed to the fact that the Bernards model considers a constant mobility not describing the real physics of the device. For more complex models, such as the one recently published by Friedlein et al., this system becomes impossible to solve analytically due to the power dependence of the source–drain bias in the model.^31^

One aspect that we neglected so far is the requirement of a high supply voltage at higher drain load to keep the same operation point at zero gate bias (see Equation (1)). As the PEDOT:PSS‐based OECT is a normally ON device and is shut down upon applying a positive gate voltage, the drain current is modulated from *I*_max_ to zero. In the same manner, the effective source–drain bias on the OECT, i.e., *V*_out_, will vary from the set operation point up to *V*_supply_. This may lead to the unfortunate situation where the applied bias gets bigger than the electrochemical potential for water hydrolysis, inevitably destroying the device.^32^, ^33^ In Figure 2c, the source–drain bias is plotted against the gate voltage for different drain load resistors. The steeper this curve is, the better the amplification. But the device gets out of the stable operation window |*V*_out_ − *V*_GS_| < 1 V at far lower gate voltage. In order to avoid any instability issues it is best to limit the input signal to low amplitudes or use a negative polarity for the gate signal, bearing in mind that this way the OECT is pushed back into the linear regime upon gating.

**Figure 3:**
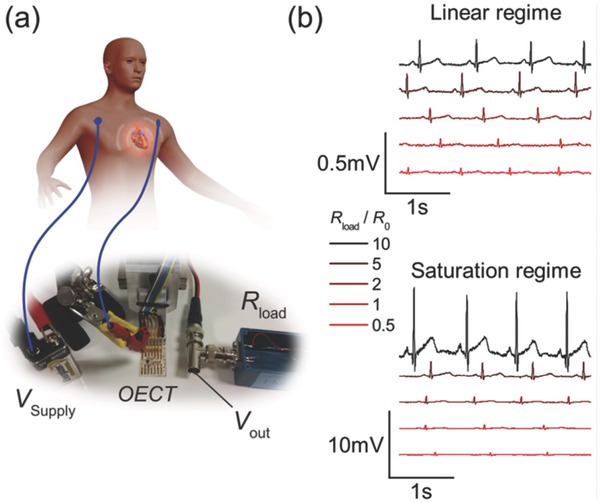
a) Schematic of the experimental setup for ECG recordings. The output voltage *V*_out_ can be directly picked up by a regular voltage recording system. The medical electrodes (blue) are placed below the clavicle to the left and to the right of the heart. The system is driven by a battery. b) ECG recordings for different drain load in both linear and saturation regime. Each curve has been recorded consecutively with the same device. The channel dimensions of the OECT are *W*/*L* = 2 and *d* = 70 nm. The amplitude of the input signal recorded from the same electrodes was of the order of ∆*V*_ECG_ ≈ 300 μV. The recordings have been taken in a low noise environment.

However, as we are interested in recording biological signals such as electrocardiograms (ECG) or electroencephalograms (EEG), and the amplitude of those signals is generally in the order of a few hundreds of microvolt, we can safely assume that at *V*_GS_ = 0 V we stay within the stable operation window of the OECT.^34^ As a proof of concept, we use the OECT‐based voltage amplifier system for measuring heart activity. For this, we place two medical electrodes below the clavicle on the left and the right side of the chest and connect them to the source and the gate of the OECT (see **Figure** **3**a).^35^ This way, the OECT is operated at *V*_GS_ = (0 V + ECG) and the heart activity is doping or dedoping the channel.^36^ The medical electrodes provide a good contact with the skin and allow for an effective gating. We note that in this configuration, the OECT is not in contact with the skin, thus acting solely as an external preamplifier. Using this kind of configuration, we are able to control the value of the drain load resistor, which would not be possible in an all integrated solution. Due to the inherent voltage conversion at the drain load resistor, we can directly connect the output signal to a regular acquisition system for electrophysiology. For comparison, we record the heart activity with the OECT operated in linear and saturation regime. Upon varying the drain load resistor, we can modify the amplification of the signal (see Figure 3b). It can be seen that operating the OECT in the linear regime does not give any substantial amplification of the input signal. At best there is a one to one transduction for high drain load. As already stated in Figure 2a, the amplification saturates for values of *R*_load_ > 5 * *R*_chan_. Nonetheless, a clear ECG signal can be seen with all relevant features of the PQRST complex (characteristic waves of the signal linked with propagation of muscle activity in the heart) for practically every value of the drain load resistor.^37^ The same holds when operating the device in the saturation regime with the difference being a much higher amplitude of the output signal and an amplification of as much as Gain_sat_ = 30 V/V for the highest recorded drain load resistor *R*_load_ = 10 * *R*_chan_. We note that the signal‐to‐noise ratio does not seem to change for the different recordings (see the Supporting Information) which can be mainly attributed to the fact that the experiment has been conducted in a low‐noise environment, inside a Faraday cage room.

## 3 Conclusion

We have successfully demonstrated the working principle of a voltage amplifier circuit based on a microfabricated organic electrochemical transistor with high transconductance using a simple resistor in series. This creates a floating voltage point that linearly depends on the drain current and can be used to transduce signals picked up by the OECT. As a proof of concept, we use the OECT‐based voltage amplifier externally in combination with medical electrodes. Characterization measurements show that a much better performance can be achieved upon driving the transistor in the saturation regime. In this case, the drain current is independent of the source–drain bias of the OECT and thus the output voltage depends directly on the input voltage at the gate of the OECT. We were able to record electrocardiographic signals by using a voltage recording system utilized for standard tests in a hospital. To our knowledge, this is the first time voltage‐to‐voltage amplifying transduction of electrophysiological activity has been demonstrated with an OECT, rendering this device directly useful in a clinical setup. Future work is ongoing to implement a gel electrolyte allowing for long‐term measurements directly on the skin to provide an active organic device that can be compared to regular electrodes.

## 4 Experimental Section

*Device Fabrication*: The devices were fabricated according to the parylene lift‐off method reported previously.^11^ Standard microscope glass slides were cleaned via sonication in an acetone and isopropyl alcohol solution and dried with nitrogen. Connection pads and interconnects were deposited through a lift‐off process using photolithographic patterning of positive photoresist (S1813) with a SUSS MJB4 broad band UV light mask aligner and MF‐26A developer. A subsequent metal deposition via evaporation of chromium (10 nm) and of gold (120 nm) and metal lift‐off using acetone defines the gold lines. A first layer of parylene C (2 μm), deposited via a SCS Labcoater 2 together with a small amount of 3‐(trimethoxysilyl)propyl methacrylate (A‐174 Silane) to enhance adhesion, acts as an insulator to prevent disturbing capacitive effects at the metal liquid interface. Subsequently, an antiadhesive layer was spin coated using a dilution of industrial cleaner (2%, Micro‐90) and a second parylene C sacrificial layer (2 μm) is deposited. To define the contact pads and the channel of the OECT, a second photolithographic patterning step using a thick positive photoresist (5 μm, AZ9260) and AZ developer is used to protect the parylene C layers from a subsequent plasma reactive ion etching step via an Oxford 80 Plasmalab plus (400 W, O_2_ = 50 sccm, CHF_3_ = 5 sccm, 8 min), at the point where no PEDOT:PSS is supposed to stay. The PEDOT:PSS solution was prepared mixing an aqueous dispersion (19 mL, Clevios PH‐1000 from Heraeus Holding GmbH) with ethylene glycol (1 mL) and dodecyl benzene sulfonic acid (50 μL), to improve conductivity and enhance film formation and sonicating for 40 min, then adding (3‐glycidyloxypropyl) trimethoxysilane (1 wt%), to prevent delamination of the film and sonicating for another 5 min. This dispersion was spincoated for a target thickness of about 90 nm (3000 rpm, 90 s) and baked (90 °C, 90 s). The PEDOT:PSS thus covers the whole glass slide and fills the “wells” defined in the second photolithographic patterning step. Peeling of the sacrificial layer and hard baking the device (125 °C, 1 h) thus define the channel width, the distance between source and drain contact defines the channel length. Immersing the devices in deionized water over night removes excess low molecular weight compounds.

*Device Characterization*: All experiments were done using a NaCl solution (100 mm) as electrolyte and a Ag/AgCl pellet (Warner Instruments) as gate electrode. The IV curves were recorded using a Keithley 2612A dual SourceMeter with customized LabVIEW software. For the precharacterization experiments with the drain load a Voltcraft R‐BOX 01 decade resistor box was connected to the OECT and a Keithley 2612A dual SourceMeter was used for the supply voltage, the output voltage was recorded using a National Instruments USB‐6251 BNC data acquisition system and customized LabVIEW software. The ECG recordings were done in a low noise room at the hospital La Timone in Marseille, France. Ambu sensor N medical Ag/AgCl electrodes with 0.95 cm diameter gel‐assisted contact area were used as standard ECG electrodes to make contact with the skin. A battery was used for the supply voltage to reduce the noise coming from building ground and the output voltage was recorded using a Braintronics voltage recording system with Brainbox EEG‐1166 amplifiers. The data were treated subsequently using a 49–51 Hz band block fast Fourier transform (FFT) filter, a 100 Hz low pass FFT filter, and a 0.05 Hz high pass FFT filter.

## Supporting information

As a service to our authors and readers, this journal provides supporting information supplied by the authors. Such materials are peer reviewed and may be re‐organized for online delivery, but are not copy‐edited or typeset. Technical support issues arising from supporting information (other than missing files) should be addressed to the authors.

Supplementary
