# Supplementary material for: Voltage Amplifier Based on Organic Electrochemical Transistor
Source: Adv Sci (Weinh). 2016 Sep 17;4(1):1600247. doi: 10.1002/advs.201600247 (PMC5238735; doi:10.1002/advs.201600247)
Supplement: Supplementary file 1 — Supplementary [file ADVS-4-0-s001.pdf]

Copyright WILEY-VCH Verlag GmbH & Co. KGaA, 69469 Weinheim, Germany, 2016.

ADVANCED SCIENCE
Open Access

Supporting Information

for *Adv. Sci.,* DOI: 10.1002/advs.201600247

Voltage Amplifier Based on Organic Electrochemical Transistor

*Marcel Braendlein, Thomas Lonjaret, Pierre Leleux, Jean-Michel Badier, and George G. Malliaras**

Copyright WILEY-VCH Verlag GmbH & Co. KGaA, 69469 Weinheim, Germany, 2013.

Supporting Information

Voltage amplifier based on organic electrochemical transistor

*Marcel Braendlein, Thomas Lonjaret, Pierre Leleux, Jean-Michel Badier, George G. Malliaras**

Marcel Braendlein
Department of Bioelectronics, Ecole Nationale Supérieure des mines
CMP-EMSE, MOC, Gardanne 13541, France
E-mail: marcel.braendlein@emse.fr

Thomas Lonjaret
Department of Bioelectronics, Ecole Nationale Supérieure des mines
CMP-EMSE, MOC, Gardanne 13541, France
&
MicroVitae Technologies, Hôtel Technologique, Meyreuil 13590, France
E-mail: thomas.lonjaret@emse.fr

Dr. Pierre Leleux
Department of Bioelectronics, Ecole Nationale Supérieure des mines
CMP-EMSE, MOC, Gardanne 13541, France
E-mail: leleux@emse.fr

Dr. Jean-Michel Badier
Institut de Neurosciences des Systèmes, Aix-Marseille Université
INS/Inserm, 13005 Marseille, France
E-mail: jean-michel.badier@univ-amu.fr

Prof. George G. Malliaras
Department of Bioelectronics, Ecole Nationale Supérieure des mines
CMP-EMSE, MOC, Gardanne 13541, France
E-mail: malliaras@emse.fr

Bernards model[1]

Here we present a derivation of an analytical expression for the gain of the OECT based voltage amplifier.

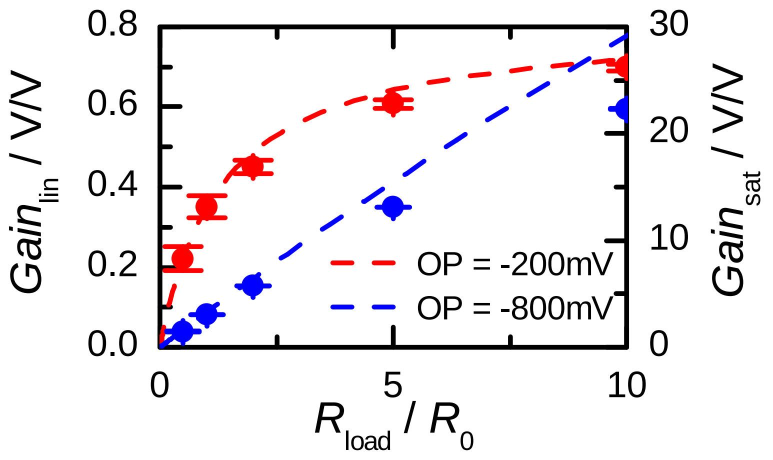

Figure S1. Voltage gain ΔV_{out} / ΔV_{GS} for different drain load resistors for an input signal of ΔV_{GS} = 1 mV in both the linear regime (red, OP_{lin} = -0.2 V) and the saturation regime (blue, OP_{sat} = -0.8 V). The channel resistance is R_0 = 213 Ω and R_0 = 513 Ω for the linear regime and saturation regime respectively. The dashed curve shows a fit using the Bernards model (see Equation S6 and Equation S10). The fit parameters are G = 5.8 mS and V_P = 0.53 V.

Linear regime

In the linear regime ($V_{out} > V_{GS} - V_P$), the drain current is given by

$$I_D = G\left(1 - \frac{V_{GS} - 0.5 \cdot V_{out}}{V_p}\right) \cdot V_{out} \quad , \qquad \text{S1}$$

where $G = q\mu p_0 WT/L$ and $V_P = qp_0T/c_d$.

Using the load line equation

$$I_D = \frac{V_{supply}}{R_{load}} - \frac{V_{out}}{R_{load}} \quad , \qquad \text{S2}$$

the drain current can be eliminated and a quadratic equation in V_{out} can be obtained (for ease of reading we denote the drain load resistor R_{load} simply with R from now on)

$$[0.5 \cdot GR] \cdot V_{out}^2 + [GRV_p - GRV_{GS} + V_p] \cdot V_{out} + [-V_pV_{GS}] = 0 \quad . \qquad \text{S3}$$

This can be solved for V_{out}:

$$V_{out} = \frac{-(GRV_p - GRV_{GS} + V_p) \pm \sqrt{(GRV_p - GRV_{GS} + V_p)^2 + 2GRV_pV_{supply}}}{GR} \quad . \qquad \text{S4}$$

Note that the negative square root in Equation S4 can be neglected as $|V_{out}| \leq |V_{supply}|$ which holds only for the positive square root.

The gain is thus given by the derivative

$$Gain_{sat} = \left|\frac{\partial V_{out}}{\partial V_{GS}}\right| = \left|1 - \frac{V_P + GR(V_P - V_{GS})}{\sqrt{(V_P + GR(V_P - V_{GS}))^2 + 2GRV_PV_{supply}}}\right| \quad \text{S5}$$

Note that the gain is smaller than one, meaning that no amplification of the signal is possible in the linear regime.

At $V_{GS} = 0$ this simplifies to

$$Gain_{sat} = \left|1 - \frac{V_P \cdot (GR + 1)}{\sqrt{V_P^2 \cdot (GR + 1)^2 + 2GRV_PV_{supply}}}\right| . \quad \text{S6}$$

__Saturation regime__

In the saturation regime ($V_{out} \leq V_{GS}$ - V_P), the drain current is given by

$$I_D = -\frac{G \cdot (V_{GS} - V_P)^2}{2V_P} , \quad \text{S7}$$

and the output voltage can be derived to

$$V_{out} = \frac{GR}{2V_P} \cdot V_{GS}^2 - GR \cdot V_{GS} + {}^1\!/_2\, GRV_P + V_{supply} . \quad \text{S8}$$

This leads to a gain of

$$Gain_{sat} = \left|\frac{\partial V_{out}}{\partial V_{GS}}\right| = \left|GR \cdot \left(\frac{V_{GS}}{V_P} - 1\right)\right| , \quad \text{S9}$$

which scales linearly with the drain load.

At $V_{GS} = 0$ the gain is directly proportional to the product of the conductance and the drain load

$$Gain_{sat} = |-GR| . \quad \text{S10}$$

Signal-to-noise analysis

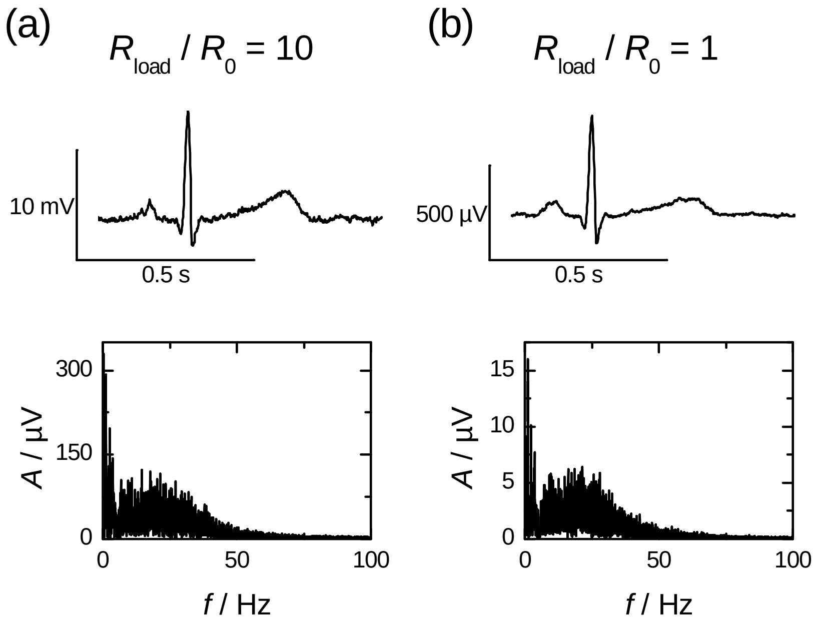

Figure S2. Comparison of ECG signal recorded for two different values of the drain load resistor, (a) R_{load} = 5.13 kΩ and (b) R_{load} = 513 Ω. The OECT is operated in the saturation regime (-0.8 V). The Fourier transform shows the same frequency content and the signal-to-noise ratio does not differ between the two measurements. The presented data is unfiltered.

[1] D. A. Bernards, G. G. Malliaras, *Adv. Funct. Mater.* **2007**, *17*, 3538.
